# Compliance with Prescription Guidelines for Glucose-Lowering Therapies According to Renal Function: Real-Life Study in Inpatients of Internal Medicine, Endocrinology and Cardiology Units

**DOI:** 10.3390/medicina57121376

**Published:** 2021-12-17

**Authors:** Laura Lohan, Florence Galtier, Thibault Manson, Thibault Mura, Audrey Castet-Nicolas, Delinger Faure, Nicolas Chapet, Florence Leclercq, Jean Luc Pasquié, François Roubille, Camille Roubille, Hubert Blain, Philippe Guilpain, Maxime Villiet, Antoine Avignon, Ariane Sultan, Cyril Breuker

**Affiliations:** 1 Clinical Pharmacy Department, CHU Montpellier, University Montpellier, 34295 Montpellier, France; l-lohan_descamps@chu-montpellier.fr (L.L.); thibaut.manson@gmail.com (T.M.); audrey-castet@chu-montpellier.fr (A.C.-N.); d.faure@hopital-clermont-lherault.fr (D.F.); n-chapet@chu-montpellier.fr (N.C.); m-villiet@chu-montpellier.fr (M.V.); 2 PhyMedExp, University Montpellier, CNRS, INSERM, 34295 Montpellier, France; f-roubille@chu-montpellier.fr (F.R.); c-roubille@chu-montpellier.fr (C.R.); a-avignon@chu-montpellier.fr (A.A.); a-sultan@chu-montpellier.fr (A.S.); 3 Endocrinology-Diabetology-Nutrition Department, University Montpellier, 34295 Montpellier, France; f-galtier@chu-montpellier.fr; 4 Clinical Research and Epidemiology Unit, CHU Montpellier, University Montpellier, 34295 Montpellier, France; Thibault.MURA@chu-nimes.fr; 5 Cardiology Department, CHU de Montpellier, University Montpellier, 34295 Montpellier, France; f-leclercq@chu-montpellier.fr (F.L.); jl-pasquie@chu-montpellier.fr (J.L.P.); 6 Department of Internal Medicine, Montpellier University Hospital, 34295 Montpellier, France; 7 Department of Internal Medicine and Geriatrics, University Montpellier, 34295 Montpellier, France; h-blain@chu-montpellier.fr; 8 Internal Medicine and Multi-Organic Diseases Department, University Montpellier, 34295 Montpellier, France; p-guilpain@chu-montpellier.fr

**Keywords:** diabetes mellitus, renal function, cardiovascular medicine, glucose-lowering drugs, prescription guidelines

## Abstract

*Background and objectives:* Renal failure is a contraindication for some glucose-lowering drugs and requires dosage adjustment for others, particularly biguanides, sulfonylureas, and inhibitors of dipeptidyl peptidase 4. In this study, we assessed adherence to prescription recommendations for glucose-lowering drugs according to renal function in hospitalized diabetic subjects. *Materials and Methods*: This prospective cohort study was carried out over a 2-year period in a university hospital. Glomerular filtration rate (GFR) was determined by averaging all measurements performed during hospitalization. Glucose-lowering drug dosages were analyzed according to the recommendations of the relevant medical societies. *Results*: In total, 2071 diabetic patients (53% hospitalized in cardiology units) were examined. GFR was <30 mL/min/1.73 m^2^ in 13.4% of these patients, 30–44 in 15.1%, 45–60 in 18.3%, and >60 in 53.3%. Inappropriate oral glucose-lowering treatments were administered to 273 (13.2%) patients, including 53 (2.6%) with a contraindication. In cardiology units, 53.1% and 14.3% of patients had GFRs of <60 and <30 mL/min/1.73 m^2^, respectively, and 179 (15.4%) patients had a contraindication or were prescribed an excessive dose of glucose-lowering drugs. *Conclusions*: We showed that the burden of inappropriate prescriptions is high in diabetic patients. Given the high number of patients receiving these medications, particularly in cardiology units, a search for potential adverse effects related to these drugs should be performed.

## 1. Introduction

The prevalence of diabetes mellitus continues to increase worldwide. Once established, diabetes can lead to several complications that reduce the quality of life and increase mortality, including retinopathy, nephropathy, neuropathy, and a variety of cardiovascular problems such as heart failure. Diabetes affects between 20% and 40% of heart failure patients [1,2]. These complications can be reduced or prevented with optimal control of blood glucose, blood pressure, and lipids. In addition, diabetes mellitus is the leading cause of chronic kidney disease (CKD). It is estimated that 20% to 40% of diabetic patients (all types included) will develop CKD [3,4]. Regarding the side effects of antidiabetic treatment, CKD increases the risk of metformin-associated lactic acidosis and hypoglycemia induced by insulin-secreting drugs when kidneys are involved in their metabolism [5,6,7].

The international therapeutic guidelines now take into account the new glucose-lowering therapy classes that are currently available, as well as the new knowledge of side effects [8,9]. Until recently, most guidelines did not recommend metformin for patients with moderate-to-severe CKD [10]. In 2016, the contraindication for CKD stages 3A and 3B was replaced by dose adjustments and glomerular filtration rate (GFR) monitoring, according to the European Medicines Agency and the US Food and Drug Administration (FDA) [11,12]. Sulfonylureas are contraindicated by most international guidelines in severe CKD, and prescription caution is recommended in moderate CKD, notably due to an increased risk of hypoglycemia [3,8]. Glipizide, glimepiride, and gliclazide may carry a lower risk for hypoglycemia compared with other sulfonylureas [8]. Inhibitors of dipeptidyl peptidase 4 (DPP-4 inhibitors) are also largely excreted by kidneys [13,14], and their dose must be adapted to the GFR level [15].

Nevertheless, little is known about how the guidelines are implemented in general practice. Studies that have investigated the use of glucose-lowering drugs according to kidney function have focused on specific populations such as outpatients and older and non-institutionalized patients [16,17,18]. Moreover, as most of these studies investigated the various therapeutic classes of antidiabetic agents and not individual drugs, specific molecules and doses have not been taken into account [19,20].

Therefore, the aim of the present study was to assess the adherence to prescription recommendations for glucose-lowering drugs (integrating molecules and dosages) of diabetic subjects at the time of their hospital admission and according to renal function.

## 2. Materials and Methods

### 2.1. Study Design, Setting and Participants

This prospective cohort study was conducted over a 2-year period from January 2016 to January 2018 in the University Hospital of Montpellier, France. Participation was proposed by clinical pharmacists to all adult patients admitted to 10 medical units (5 internal medicine units including 1 specialized in geriatrics, 2 endocrinology units, and 3 cardiology units), regardless of the reason for admission. This study is an ancillary study of the “Incidence and Risk Factors of Drug-Related Problems Detected in Inpatients” study (DRPINPAT study, ClinicalTrials.gov NCT03476733).

### 2.2. Medication History Cohort Design

A pharmaceutical team (including a senior pharmacist and/or a resident, as well as pharmacy students) conducted a medication reconciliation process within 24 h of admission or on the first working day following admission to the unit. The medication reconciliation process was conducted according to a validated protocol previously described [21]. Briefly, the best possible medication history (BPMH), which corresponds to the list of all medications taken by the patient, including prescription medication and over-the-counter drugs, has to be based on at least three sources of information.

### 2.3. Data Collection

**Figure 1:**
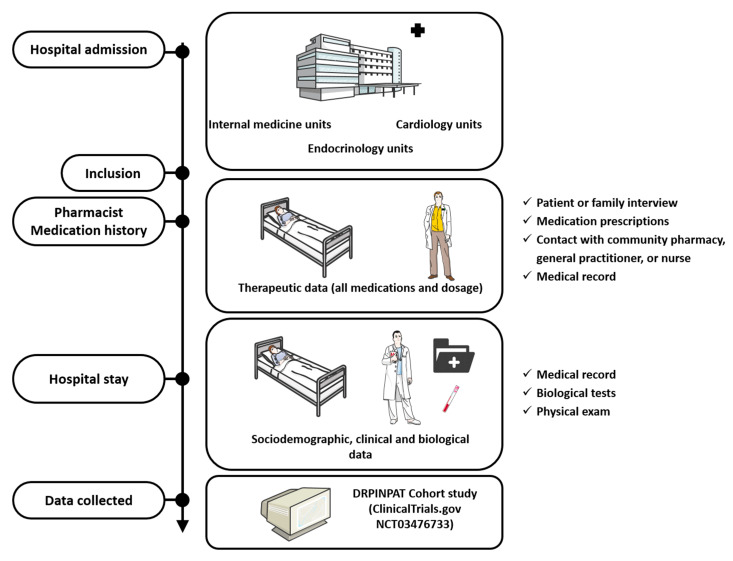
Data collection process.

Data included demographic (age, gender), clinical (number and type of antidiabetic treatment, admission unit), and biological information (GFR, *Glycated haemoglobin* A1C (HbA1c)). They were retrieved from the medical record and the patient’s report of the medications taken the day before hospitalization (names and daily doses) through the medication reconciliation process (Figure 1). The GFR was calculated according to the Chronic Kidney Disease Epidemiology Collaboration (CKD-EPI) formula and by averaging all the measurements performed during hospitalization. It was expressed in mL/min/1.73 m^2^. In the case of kidney function with GFR < 30 mL/min, either a previous record of creatinine dosage or a previous diagnosis of chronic renal failure was searched for in the medical record. For HbA1c, either dosages performed during the inpatient stay or values obtained in the past 6 months at the same site were used. All dosages (GFR and HbA1c) were performed in the same laboratory of the Montpellier University Hospital. Diabetes medication daily doses (biguanides, sulfonylureas, glinides, glucagon-like peptide-1 receptor (GLP-1) agonists, DPP-4 inhibitors) were classified into three categories: appropriate dose, excessive dose, or contraindication (use not recommended). For each drug, the CKD-appropriate dose or contraindication was based on the manufacturer’s labeling and the recommendations of the relevant medical societies [8,9], summarized in Table 1.

### 2.4. Statistical Analysis

Patient characteristics and types of medication were described with proportions for categorical variables and with means ± standard deviations (SD) for quantitative variables. These characteristics were compared between appropriate and inappropriate (excessive dose and contraindication) doses for biguanides, sulfonylureas, and DPP-4 inhibitors. They were compared according to the GFR with the Student’s *t*-test or the Mann–Whitney U-test for continuous variables, and with the Chi-square or Fisher’s exact test for categorical variables.

Factors associated with an excessive dose for biguanides, sulfonylureas (patients with GFR between 30 and 60 mL/min/1.73 m^2^), and DPP-4 inhibitors (patients with GFR below 60 mL/min/1.73 m^2^) were first determined using the Student’s *t*-test or the Mann–Whitney U-test for continuous variables, and with the Chi-square or Fisher’s exact test for categorical variables. We then assessed these associations using univariate and multivariable logistic regression analyses. The variables that were entered in the multivariable model were those with a *p*-value < 0.2 in the univariate analysis. Only the factors with a multivariable *p*-value < 0.1 were finally retained in the model using a backward stepwise selection procedure.

Statistical analyses were performed at the conventional two-tailed α level of 0.05, using SAS version 9.1 (SAS Institute, Cary, NC, USA).

### 2.5. Ethical Statement

All subjects gave their informed consent for inclusion before they participated in the study. Our study follows the World Medical Association’s Declaration of Helsinki and was approved by our hospital Institutional Review Board (2018_IRB-MTP_04-14).

## 3. Results

### 3.1. Characteristics of the Study Population

**Figure 2:**
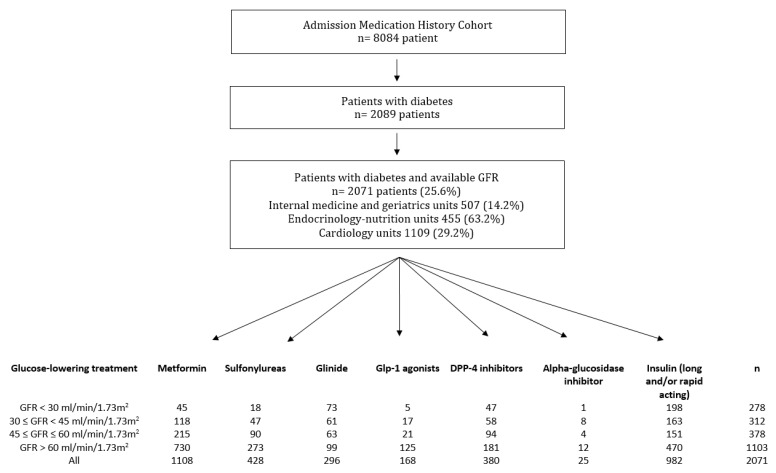
Flowchart of the study population. Data are n. GFR, glomerular filtration rate; DPP-4, dipeptidyl peptidase-4; GLP1, glucagon-like peptide-1 receptor agonist.

In total, 8084 patients were evaluated. On admission, 2089 (25.8%) were receiving glucose-lowering treatment. Among them, 2071 (99% of those receiving a glucose-lowering treatment) had an available evaluation of GFR and were included in our study cohort (Figure 2). Thus, 14.2% of the patients hospitalized in internal medicine and geriatrics (507/3567) units, 63.2% in endocrinology-nutrition units (455/720), and 29.2% (1109/3797) in cardiology units were living with diabetes. The median number of GFR measurements per patient to assess renal function was 3 [1,2,3,4,5,6,7], and the median standard deviation was 4.6 mL/min/1.73 m^2^ [2.5–4.8]. In total, 526 (25%) patients had only one measurement of GFR, including 365 (69%) hospitalized in a weekly planned hospitalization unit for disease evaluation. The mean age was 71.0 years (SD 13.9), 59.4% were men, and 13.4% had a GFR < 30 mL/min/1.73 m^2^. Table 2 describes the characteristics of the study population according to their GFR. Patients with low GFR were older and had a higher number of home treatments (Table 2). In total, 57.0% of diabetes patients hospitalized in internal medicine and geriatrics units, 19.8% in endocrinology-nutrition units, and 53.1% in cardiology units had a GFR below 60 mL/min/1.73 m^2^. Most patients received either one (39.8%) or two (36.9%) glucose-lowering drugs, including insulin.

Biguanides and sulfonylureas were more frequently used in patients with normal renal function, whereas glinides and insulin were more frequent when renal function was impaired. Among metformin-treated patients, daily doses were not different across GFR categories.

### 3.2. Inappropriate Prescription of Glucose-Lowering Treatment According to the Glomerular Filtration Rate

A total of 273 (13.2%) patients had at least one inappropriate oral glucose-lowering treatment prescription, including 230 (11.1%) with excessive doses and 53 (2.6%) with contraindication (45 biguanide and 18 sulfonylurea treatments) at the time of admission. Ten patients with inappropriate oral glucose-lowering treatment prescriptions had both an excessive dose for one treatment and a contraindication for another. All classes were concerned: biguanides (15.0% excessive doses and 4.0% contraindicated), DPP-4 inhibitors (15.0% excessive doses), sulfonylureas (2.1% excessive doses and 4.2% contraindicated), and glinides (4.1% excessive doses). In addition, the proportion of subjects with an inappropriate dose increased with the severity of renal impairment (Table 3). No overdosages were observed in patients with a GFR > 60 mL/min/1.73 m^2^.

We looked for previous documentation of chronic renal disease in the 53 patients with contraindicated treatment (35 biguanides, 8 sulfonylureas, and 10 both biguanides and sulfonylureas). Severe chronic renal failure (GFR < 30 mL/min/1.73 m^2^) was clearly documented in 27 of the patients (51%), whereas chronic renal failure without a recent GFR estimation was recorded for 6 (11.3%). When compared to the last available assessment, worsening of chronic renal failure (between 30 and 45 before hospitalization) was noted for 17 (32.1%) patients. One did not have chronic renal failure prior to hospitalization, and his low GFR was due to acute renal failure following chemotherapy. No data on pre-existing renal failure were available for two of the patients. Analysis of other chronic treatments in these 53 patients showed that 45 (84.9%) of them were also being treated with at least one nephrotoxic drug (e.g., angiotensin-converting-enzyme inhibitor, diuretic, angiotensin-2 receptor antagonist). A venous lactate acid assay was available for 19/45 (42.2%) metformin-treated patients with a contraindication. Nine of them had increased plasma lactate concentrations, including four with values above 5 mmol/L. The presence or absence of hypoglycemia in patients treated with a sulfonylurea and presenting a contraindication was not documented in 96% of cases (only one patient with a notion of hypoglycemia). Finally, in 86% of cases, contraindicated treatments were stopped during hospitalization and not prescribed at discharge (92.5% of biguanide treatments and 75% of sulfonylureas).

### 3.3. Variables Associated with an Excessive Daily Dose of Glucose-Lowering Treatment 

In the subgroup of patients requiring dose adjustments of biguanides (GFR between 30 and 60 mL/min/1.73 m^2^), 49.8% had excessive daily doses, and in the subgroup requiring dose adjustments of sulfonylureas, 7% had excessive daily doses (Table 4). In the subgroup of patients requiring dose adjustments of DPP-4 inhibitors (GFR < 60 mL/min/1.73 m^2^), 28.6% had excessive daily doses. In these three subgroups, there were no contraindicated treatments. In cardiology units, 53.7% (108/201), 4.9% (4/77), and 32.3% (40/124) of the patients had excessive doses of biguanides, sulfonylureas, and DPP-4 inhibitors, respectively.

Patients with an excessive dose of biguanides were more often men, were younger, had higher levels of HbA1c, and had received a higher number of glucose-lowering medications. Patients with an excessive dose of sulfonylureas were more frequently co-treated with insulin, notably basal insulin, and had received a higher number of glucose-lowering medications. Patients with an excessive dose of DPP-4 inhibitors had lower GFR values.

As presented in Table 5, the multivariable analysis showed that lower ages were associated with the risk of receiving an excessive daily dose of biguanides. For sulfonylureas, insulin treatment was associated with the risk of receiving an excessive daily dose. A lower GFR value and non-insulin treatment were associated with an excessive dose of DPP-4 inhibitors.

## 4. Discussion

Our study revealed that, on admission to various medical units, 13.2% of the patients with diabetes and treated by glucose-lowering drugs (i.e., 26% of the entire cohort) were receiving either contraindicated or excessive doses of glucose-lowering treatment according to their renal function. This risk was particularly high for metformin (19.0%), DPP-4 inhibitors (15.0%), and sulfonylureas (6.3%).

Only a few reports have emphasized this discrepancy between therapeutic guidelines and real-life management in patients with diabetes and CKD [16,19,22,23].

Several explanations might account for this poor adherence to prescription guidelines: (i) insufficient screening for CKD; (ii) lack of knowledge among prescribers of the GFR thresholds that trigger dose adjustments; (iii) a degree of therapeutic inertia, with long-used drugs not being questioned soon enough, furthering the progression of renal disease. One of the limitations of our data is that we cannot offer any conclusion regarding the impact of these hypotheses on our population. Nevertheless, we have some degree of insight into the possible mechanisms.

A lack of renal monitoring was highlighted in 2007 in the ENTRED study [24]. This study showed that only 80% of type 2 diabetic outpatients had an evaluation of serum creatinine once a year, and less than one third had their albumin or protein urine excretion rate measured at least annually. Yet, after a one-year follow-up, 15.4% of them showed deterioration in their kidney function [20]. The absence of renal function monitoring was associated with an increase in cardiovascular/renal events and mortality (odds ratio (95%CI), 1.32 (1.07–1.64)), in both type 1 and type 2 diabetes [25]. In our cohort, previous chronic renal insufficiency, with a GFR below 30 or close to it, had been reported in most of the patients with at least one contraindication.

Our results are comparable to those of the OREDIA study, which was carried out in 2012 in a French population of 3704 type 2 diabetes mellitus outpatients with CKD. In this cohort, the detection of CKD was fairly good, whereas the adjustment of the antidiabetic treatment to the CKD level was insufficient: only 34% of the patients with severe CKD had an appropriate drug adjustment [16]. Similarly, Christiansen et al. [23] found that 44% and 62% of diabetic patients in, respectively, Denmark and the UK continued to take metformin in spite of a decline in GFR to below 30 mL/min/1.73 m^2^. In these two cohorts, as in ours, it is impossible to tell whether this was due to insufficient knowledge or to therapeutic inertia.

Poor adherence to therapeutic recommendations was associated with the following: lower ages for biguanides, insulin treatment for sulfonylureas, and lower GFR values and non-insulin for DPP-4 inhibitors. For all medications, inappropriate prescriptions were found for both genders and in patients hospitalized in all three types of units. The OREDIA study showed that poor adherence to therapeutic recommendations occurred significantly more often in patients followed by a general practitioner (33%) than in those followed by a diabetologist (85%). We did not record this data precisely, but insulin-treated patients and patients hospitalized in endocrinology-nutrition units were more often followed by a diabetologist.

The consequences of inappropriate antidiabetic treatment depend on the class. Metformin accounted for most of the inappropriate prescriptions in our study. The incidence of lactic acidosis among metformin-treated patients is very low, even in stable CKD stage 3, which may lead prescribers to overlook this risk [6,26,27]. Indeed, in our cohort, plasma lactate concentrations were above normal in 47% of the chronic kidney failure patients in whom it was tested, including 44% with values above 5 mmol/L.

For sulfonylureas, the risk of hypoglycemia is significantly increased in renal insufficiency [28]. Hypoglycemic episodes that are not readily explained by conventional factors (skipped or irregular meals, unaccustomed exercise, alcohol ingestion, etc.) may be due to excessive doses of the drugs used to treat diabetes. Moreover, inpatient hyperglycemia has been associated with prolonged hospital stays and numerous adverse outcomes, including mortality [29]. Thus, the American Diabetes Association and the Endocrine Society workgroup on hypoglycemia and diabetes emphasized that clinicians and educators need to assess the risk of hypoglycemia at every visit with patients treated with insulin and insulin secretagogues [29]. In a previous study, we found that patients generally lacked awareness of the risk of hypoglycemia [30]. In the present study, we found that hypoglycemia was not sufficiently investigated in patients with renal insufficiency and with dosages that were are too high. Interestingly, there seems to have been a considerable change in sulfonylurea prescriptions in recent years. Indeed, the use of sulfonylurea in subjects with severe renal impairment was only 6.5% in our study, but as high as 20% and 18.1% in the Penfornis et al. and RIACE studies, respectively [16,19]. In the ENTRED study, published more than 10 years ago, there was no difference in sulfonylurea treatment according to renal function in people with type 2 diabetes mellitus: sulfonylureas were prescribed in 49%, 51%, 52%, and 56% of patients with normal, mildly decreased, moderately decreased, and severely decreased CKD, respectively [20]. Our data suggest that this results from a switch from sulfonylureas to DPP-4 inhibitors in patients with CKD <60 mL/min/1.73 m^2^, as we observed that DPP-4 inhibitor prescriptions are the highest for GFR between 30 and 60 mL/min/1.73 m^2^. The frequency of use of DPP-4 inhibitors in our study is close to that described in the study from Min et al. (19.6% and 17.6% in people with moderate and severe renal deficiency, respectively) [22].

In the cardiology units, 29.2% (n = 1109) of the patients were living with diabetes, which represented more than half of all patients in this study (53.5%). More than 50% of the diabetic patients in the cardiology units had impaired renal function with CKD of less than 60 mL/min/1.73 m^2^, and 14.3% of the patients had a GFR of less than 30 mL/min/1.73 m^2^. Eighteen cardiology patients had a contraindication for glucose-lowering drugs, and 152 had an excessive dose. These results underline the importance of the medical management of diabetes, which is a comorbidity frequently found in patients hospitalized in cardiology units. In our study, we found no significant differences in terms of excessive doses of biguanides, sulfonylureas, or DPP-4 inhibitors between the care units. However, due to their high representativeness, more excessive doses were found in cardiology units. For several years, the management of diabetes in patients with cardiovascular disease has been a priority and is included in the recommendations on diabetes care from most of the relevant medical societies [31]. Moreover, some classes of glucose-lowering drugs, such as DPP-4 inhibitors, sodium-glucose cotransporter-2 inhibitors (SGLT2i), and GLP1 agonists, have demonstrated significant benefits in reducing major adverse cardiovascular events, heart failure hospitalization, and the progression of CKD [31,32,33,34,35,36,37]. Finally, we have highlighted in the chronic treatments of patients with a contraindicated antidiabetic treatment a significant proportion of nephrotoxic drugs. This observation should lead the prescriber to adapt the monitoring of renal function and, if possible, to adapt the chronic treatment in these patients.

Several limitations of our study must be acknowledged. The monocentric nature of our study might limit the generalizability of our results. However, various departments were considered, and inclusions were made, regardless of the reason for hospital admission. We did not evaluate the chronic nature of renal failure in the entire sample. Concerning sulfonylureas, a dose reduction is recommended for some molecules [8,9], but the correct dosages according to the GFR are not clearly specified. As a result, we took into account the usual or maximal dosages, which may have led to underestimated results. In addition, we could not determine how long the treatments had been inappropriate. Furthermore, we did not include SGLT2i in our analysis since it was not available at that time in France. However, we observed that nearly half the patients in our cohort had a GFR < 60 mL/min/1.73 m^2^, and 28.5% had a GFR < 45 mL/min/1.73 m^2^, which limits the introduction of this drug class and requires its discontinuation for type 2 diabetes. This class of drugs also requires monitoring of the renal function, and studies evaluating compliance with these recommendations should be conducted. Finally, we did not investigate side effects like hypoglycemia in patients receiving inadequate doses of glucose-lowering treatment. Nevertheless, key strengths of the present study include: (i) the large sample size, with a broad age range, all stages of kidney dysfunction, and recruitment from different medical units; (ii) the assessment of kidney function using multiple GFR measurements throughout hospitalization, enabling us to account for the fluctuations in renal function, especially at hospital admission; (iii) the analysis of all glucose-lowering molecules and dosages.

We should bear in mind that renal function can fluctuate and that iterative evaluations are thus mandatory during follow-up. It can be assumed that many inappropriate doses are linked to past prescriptions that have not been reassessed. This is a particularly important issue for patients with advanced and unbalanced diabetes who already have impaired renal function and high doses of glucose-lowering drugs. The pharmaceutical team played an important role in this context. The recommendations of medical societies should be better clarified and communicated to improve their implementation.

## 5. Conclusions

We have demonstrated that: (i) CKD monitoring is greatly lacking in diabetic patients, even among inpatients; (ii) the burden of inappropriate prescription is high in diabetic patients with a GFR below 60 mL/min/1.73 m^2^; (iii) cardiology units have a high number of diabetic patients with impaired renal function and inappropriate prescriptions. Given the high number of patients receiving these medications, a search for potential adverse effects related to these drugs should be performed.

## Figures and Tables

**Table 1:** Guidelines for adjustments in diabetes treatment according to renal function.

Class	Drug	Contraindication (GFR mL/min/1.73 m^2^)	Dose Adjustment (GFR mL/min/1.73 m^2^)
Biguanides	Metformin	<30	≥30–<60 Dose ≤ 1500 mg/day *
Sulfonylureas	Glimepiride	<30	≥30–<60 Dose < 6 mg/day **
	Gliclazide	<30	None
	Glibenclamide	<30	≥30–<60 Dose < 15 mg/day **
Glinides	Repaglinide	None	<30 Dose < 12 mg/day **
DPP-4 inhibitors	Vildagliptin	None	<60 Dose ≤ 50 mg/day *
	Sitagliptin	None	<45 Dose ≤ 50 mg/day ** (30–44) Dose = 25 mg/day ** (<30)
	Saxagliptin	<15	≥15–<60 Dose ≤ 2.5 mg/day *
GLP-1 agonists	Dulaglutide	<30	None
	Liraglutide	<15	None
	Exenatide immediate release	<30	≥30–<50 Dose ≤ 2.5 mg/day *
SGLT2i #	Dapagliflozin	<45	<60 should not be initiated **
	Empagliflozin	<45	<60 initiation not recommended ** ≥45–<60 Dose 10 mg/day **
	Canagliflozin	<45	<60 initiation not recommended ** ≥45–<60 Dose 100 mg/day **
	Ertugliflozin	<30	<60 initiation not recommended ** Continued use not recommended with persistent GFR 30–60 mL/min/1.73 m^2^

GFR, glomerular filtration rate; DPP-4, dipeptidyl peptidase-4; GLP1, glucagon-like peptide-1 receptor agonist; SGLT2i, sodium-glucose cotransporter-2 inhibitors. * According to the Francophone Diabetology Society [9]. ** According to manufacturer’s labeling. # Not evaluated.

**Table 2:** Characteristics of the study population according to the glomerular filtration rate.

GFR Categories (mL/min/1.73 m^2^)	All	<30	30–44	45–60	>60	*p*
n (%)	2071 (100)	278 (13.4)	312 (15.1)	378 (18.3)	1103 (53.3)	
Age (years)	71.0 ± 13.9	78.2 ± 11.6	77.2 ± 10.2	75.2 ± 10.5	65.9 ± 14.3	<0.001
Gender–Male	1230 (59.4)	141 (50.7)	180 (57.7)	230 (60.9)	679 (61.6)	0.009
HbA1c (%) *	7.8 ± 1.6	7.4 ± 1.1	7.5 ± 1.4	7.4 ± 1.4	8.0 ± 1.8	<0.001
GFR (mL/min/1.73 m^2^)	63.2 ± 27.4	20.2 ±6.9	37.8 ±4.3	52.4 ±4.5	84.9 ± 15.0	<0.001
Admission units						<0.001
Internal medicine and geriatrics	507 (24.5)	99 (35.6)	88 (28.2)	102 (27.0)	218 (19.8)	
Endocrinology-nutrition	455 (22.0)	20 (7.2)	22 (7.1)	48 (12.7)	365 (33.1)	
Cardiology	1109 (53.5)	159 (57.2)	202 (64.7)	228 (60.3)	520 (47.1)	
Number of treatments on admission	9.6 ± 4.0	11.5 ± 3.9	10.8 ± 3.5	9.9 ± 3.5	8.6 ± 4.0	<0.001
Number of glucose-lowering treatments on admission						<0.001
1	824 (39.8)	94 (33.8)	129 (41.4)	162 (42.9)	439 (39.8)	
2	765 (36.9)	143 (51.4)	134 (42.9)	129 (34.1)	359 (32.6)	
3	350 (16.9)	38 (13.7)	34 (10.9)	62 (16.4)	216 (19.6)	
≥4	132 (6.4)	3 (1.1)	15 (4.8)	25 (6.6)	89 (8.1)	

Data are the mean ± SD, or n (%); HbA1c, hemoglobin A1c; GRF, glomerular filtration rate. * 661 missing data items.

**Table 3:** Inappropriate prescription (excessive dose and contraindication) of glucose-lowering treatment according to glomerular filtration rate categories.

GFR Categories (mL/min/1.73 m^2^)	All	<30	30–44	45–60	>60
Biguanides	211/1108 (19.0)	45/45 (100)	58/118 (49.2)	108/215 (50.3)	0/730 (0)
Sulfonylureas	27/427 (6.3)	18/18 (100)	2/47 (4.3)	7/90 (7.8)	0/273 (0)
Glinides	12/296 (4.1)	12/73 (16.4)	0/61 (0)	0/63 (0)	0/99 (0)
GLP1 agonists	0/168 (0)	0/5 (0)	0/17 (0)	0/21 (0)	0/125 (0)
DPP-4 inhibitors	57/380 (15.0)	21/47 (44.7)	31/58 (53.4)	5/94 (5.3)	0/181 (0)

Data are presented as n/category size (%). GFR, glomerular filtration rate; DPP-4, dipeptidyl peptidase-4; GLP1, glucagon-like peptide-1 receptor agonist.

**Table 4:** Characteristics of the study population according to dose appropriateness (excessive dose) in patients with GFR between 30 and 60 mLmin/1.73 m^2^ for biguanides and sulfonylureas and with GFR < 60 mL/min/1.73 m^2^ for DPP-4 inhibitors on admission.

	Biguanides (n = 333)			Sulfonylureas (n = 137)			DPP-4 Inhibitors (n = 199)		
	Appropriate (n = 167)	Excessive (n = 166)	*p*	Appropriate (n = 128)	Excessive (n = 9)	*p*	Appropriate (n = 142)	Excessive (n = 57)	*p*
Age (years)	75.9 (±10.7)	73.2 (±9.9)	0.011	77.1 (±10.2)	72.0 (±7.9)	0.089	77.1 (±10.4)	78.5 (±9.0)	0.47
Gender–Male	56.3	69.3	0.014	65.6	44.4	0.28	63.4	68.4	0.50
HbA1c (%)	7.1 (±1.2)	7.51 (±1.4)	0.033	7.4 (±1.5)	8.5 (±1.8)	0.10	7.3 (±1.2)	7.4 (±1.6)	0.84
GFR	47.3 (±8.4)	48.0 (±7.8)	0.50	47.8 (±8.1)	49.4 (±6.5)	0.65	43.8 (±12.8)	33.8 (±10.1)	<0.001
Units			0.15			0.016			0.50
Internal medicine/geriatrics	56 (33.5)	40 (24.1)		39 (30.5)	1 (11.1)		45 (31.7)	14 (24.6)	
Endocrinology-nutrition	18 (10.8)	18 (10.8)		12 (9.4)	4 (44.4)		13 (9.2)	3 (5.3)	
Cardiology	93 (55.7)	108 (65.1)		77 (60.2)	4 (44.4)		84 (59.2)	40 (70.2)	
Number of treatments	10.1 (±3.3)	9.9 (±3.5)	0.65	10.1 (±3.3)	10.8 (±2.2)	0.56	10.8 (±3.6)	10.6 (±3.9)	0.65
Number of glucose-lowering treatments at home			0.015			0.009			0.12
1	78 (46.7)	50 (30.1)		42 (32.8)	0 (0.0)		18 (12.7)	14 (24.6)	
2	45 (26.9)	61 (36.7)		50 (39.1)	2 (22.2)		53 (37.3)	22 (38.6)	
3	30 (18.0)	33 (19.9)		20 (15.6)	4 (44.4)		50 (35.2)	17 (29.8)	
≥4	14 (8.4)	22 (13.2)		16 (12.5)	3 (33.3)		21 (14.8)	4 (7.0)	
Insulin treatment–Yes	39 (23.3)	51 (30.7)	0.13	24 (18.7)	6 (66.7)	0.004	57 (40.1)	15 (26.3)	0.067
Basal insulin	33 (19.8)	43 (25.9)	0.18	21 (16.4)	6 (66.7)	0.002	50 (35.2)	13 (22.8)	0.089
Prandial insulin	16 (9.6)	22 (13.2)	0.29	5 (3.9)	1 (11.1)	0.34	25 (17.6)	5 (8.8)	0.11

Data are presented as mean ± SD, or n (%) HbA1c, hemoglobin A1c; GRF, glomerular filtration rate; DPP-4, dipeptidyl peptidase-4.

**Table 5:** Multivariate analysis of variables associated with excessive daily doses of biguanides, sulfonylureas, or DPP-4 inhibitors.

	Biguanides		Sulfonylureas		DPP-4 Inhibitors	
	OR IC95%	*p*	OR IC95%	*p*	OR IC95%	*p*
Age	0.96 (0.93–0.99)	0.007	1.08 (0.08–14.13)	0.95	-	-
Gender–Female vs. Male	0.80 (0.44–1.44)	0.46	-	-	-	-
HbA1c	1.2 (0.91–1.49)	0.23	3.30 (0.37–29.24)	0.28	-	
GFR	-	-	-	-	0.93 (0.90–0.96)	<0.001
Internal medicine/geriatrics vs. cardiology units	0.56 (0.30–1.05)	0.070	1.01 (0.94–1.10)	0.72	-	-
Endocrinology-nutrition vs. cardiology units	0.46 (0.19–1.14)	0.093	1.23 (0.69–2.19)	0.48	-	-
Number of antidiabetic treatment						
1	1		-	-	1.37 (0.53–3.51)	0.51
2	1.92 (0.94–3.91)	0.074	-	-	1	
3	1.80 (0.65–4.99)	0.26	-	-	1.17 (0.46–2.95)	0.74
4	1.74 (0.50–5.99)	0.38	-	-	2.16 (0.45–10.36)	0.33
Insulin treatment–Yes vs. No	1.09 (0.45–2.61)	0.85	14.18 (1.39–144.70)	0.025	0.26 (0.09–0.78)	0.016

OR, odds ratio; CI, confidence intervals, HbA1c, hemoglobin A1c; GRF, glomerular filtration rate; DPP-4, dipeptidyl peptidase-4.

## Data Availability

The data presented in this study are available upon request from the corresponding author. The data are not publicly available due to ethical restrictions.
